# Annual Announcements of Medical Lectures

**Published:** 1845-09

**Authors:** 


### ANNUAL ANNOUNCEMENTS OF MEDICAL LECTURES.

In addition to those already acknowledged in a previous number, we have received the annual circulars of the following institutions, besides one or two more which are by some accident mislaid: Transylvania University, at Lexington, Kentucky; Hampden Sidney College, at Richmond, Virginia; Pennsylvania College, at Philadelphia ; Medical Department of the University of New York at N. Y ; Willoughby Medical College, at the village of Willoughby, on the southern shore of Lake Erie ; Laporte University, at Laporte, Indiana. In the midst of so much competition, it is gratifying to find in several of these publications the assurances of *unexampled success.* Each finds occasion for exultation—one in the numerous diseases, and scarcely less numerous deaths, which so bountifully supply its clinics and dissecting room, *in consequence of being situated in a large city ;* another from the healthfulness and freedom from exposure to the temptations and vices of a *large city*, because *it is a country school* ; a third felicitates itself on the presumed benefit of the faculty being *self nominated and self governed ;* and a fourth finds advantages *in the peculiar institutions* which surround it, and the opportunities afforded for learning *sectional medicine*, and perpetuating *sectional feelings.* &c. &c. In all, we discover no lack of the natural yearnings after success, whatever deficiency there may be evinced in some of the dignity which makes success honourable.

